# Entropy and Information Inequalities

**DOI:** 10.3390/e22030320

**Published:** 2020-03-12

**Authors:** Varun Jog, James Melbourne

**Affiliations:** 1 Department of Electrical and Computer Engineering, University of Wisconsin-Madison, Madison, WI 53706, USA; 2 Department of Mathematical Sciences, University of Delaware, Newark, DE 19716, USA; jamescmelbourne@gmail.com

Entropy and information inequalities are vitally important in many areas of mathematics and engineering. In this special issue, we solicited contributions that showcase the wide applicability of information inequalities and serve as a repository of mathematical tools and techniques needed to prove information inequalities. The papers we received span various areas of mathematics, including information theory, geometry, functional analysis, hypothesis testing, and estimation theory. We are confident that this special issue will lead to an exchange of ideas between different areas of mathematics and foster new interdisciplinary research at the boundary between these areas. The ten submissions we received may be broadly divided into four categories: (1) Inequalities for discrete domains; (2) Inequalities in functional analysis; (3) Geometry-inspired inequalities; and (4) Miscellaneous topics.

- Inequalities for discrete domains:
  The paper by Abbe, Li, and Madiman [1] explores entropy inequalities for sums and differences of random variables on cyclic groups. This paper also shows applications in the design of polar codes on non-binary alphabets. In a completely different interpretation of “discrete”, the paper by Harremoës [2] explores entropy inequalities for random variables with functional dependencies that are expressed as lattices. In particular, the paper explores when Shannon-type inequalities are sufficient to completely characterize the entropy region.
- Inequalities in functional analysis:
  The Poincaré and log-Sobolev inequalities are crucial tools in probability and functional analysis. The paper Shikegawa [3] explores the exponential rate of convergence of Markov semigroups assuming the log-Sobolev inequality holds. Schlichting [4] investigates Poincaré and log-Sobolev constants in mixture distributions, when the components of the mixtures are assumed to satisfy the Poicaré and log-Sobolev inequalities. Liu, Courtade, Cuff, and Verdú [5] show how to combine the Brascamp-Lieb and Barthe inequalities from functional analysis into a single entropy inequality. Sason [6] proves new inequalities for *f*-divergences—generalizations of the well-known Kullback–Liebler divergence—and shows applications to hypothesis testing.
- Geometry-inspired inequalities:
  The paper by Marsiglietti and Kostina [7] shows new entropy inequalities for the entropy of log-concave random vectors using ideas from convex geometry. Hao and Jog [8] derive new volume and surface area inequalities in geometry using information theoretic inequalities.
- Miscellaneous:
  Mossel and Ohannessian [9] show the impossibility of learning rare events without making distributional assumptions. Their paper has an explicit construction and is relevant to estimating the “missing mass” in distribution estimation. Gu, Zha, and Yu [10] contribute to the area of rough random theory by proving basic probabilistic inequalities in this setting.
